# Stimulation of Subthalamic Nuclei Restores a Near Normal Planning Strategy in Parkinson’s Patients

**DOI:** 10.1371/journal.pone.0062793

**Published:** 2013-05-03

**Authors:** Giovanni Mirabella, Sara Iaconelli, Nicola Modugno, Giorgio Giannini, Francesco Lena, Gianpaolo Cantore

**Affiliations:** 1 Department of Neuroscience, Istituto Neurologico Mediterraneo Neuromed, Pozzilli (IS), Italy; 2 Department of Physiology and Pharmacology ‘V. Erspamer’, University of Rome La Sapienza, Rome, Italy; University of Toronto, Canada

## Abstract

A fundamental function of the motor system is to gather key information from the environment in order to implement behavioral strategies appropriate to the context. Although several lines of evidence indicate that Parkinson’s disease affects the ability to modify behavior according to task requirements, it is currently unknown whether deep brain stimulation (DBS) of the subthalamic nucleus (STN) affects context-related planning. To explore this issue, we asked 12 Parkinson’s patients with bilateral STN DBS and 13 healthy subjects to execute similar arm reaching movements in two different paradigms: go-only and countermanding tasks. In the former task patients had to perform speeded reaching movements to a peripheral target. In contrast, in the countermanding task participants had to perform the same reaches unless an infrequent and unpredictable stop-signal was shown during the reaction time (RT) indicating that they should withhold the ongoing action. We compared the performance of Parkinson’s patients in different DBS conditions. We found that patients with both DBS-ON behaved similarly to healthy subjects, in that RTs of no-stop trial increased while movement times (MTs) decreased with respect to those of go-only-trials. However, when both DBS were off, both RTs and MTs were longer in no-stop trials than in go-only trials. These findings indicate that bilateral DBS of STN can partially restore the appropriate motor strategy according to the given cognitive contexts.

## Introduction

Parkinson’s disease (PD) patients show great impairments when they face increasing cognitive and motor task demands. For instance, PD patients take longer to perform a given movement within a sequence than to perform the same movement alone [1], they show longer pauses between successive movement segments [2]–[5] and they also show slower movement in the later elements of a sequence [2], [6] than do healthy subjects.

A plausible reason to explain these findings is that PD affects the ability to plan and change behavior according to task contingencies. Classically, changes in planning have been associated with changes in RT length (e.g. [7]–[9]); however, it has been shown that, under certain conditions, subjects might adopt a strategy whereby programming continues during movement execution (e.g. [10], [11]).

Online planning strategies have been shown in PD patients by Leis et al. [12], exploiting a precue paradigm. In this task, a cue predicted the correct direction for the execution of a reaching movement in 80% of the cases (valid condition) and the wrong direction in the remaining 20% (invalid condition). In the invalid condition the target appeared at the uncued location, and to make the appropriate movement subjects needed to modify the action they were preparing. Leis et al. [12] found that PD patients exhibited longer RTs than healthy subjects, although RTs increased similarly in the two groups for the invalid condition. Nonetheless, unlike controls, patients showed a substantial slowing and variability of MTs when they had to change a pre-planned action. Thus it appears that, during the invalid condition, PD patients react normally to the appearance of the target, but when an unexpected change of the planned action is required the motor plan has to be completed during the execution phase.

Recently, Mirabella, Pani & Ferraina [13] tackled the issue of adaptive changes of motor planning from a novel perspective. They compared RTs and MTs of reaching movements executed either during an RT task, in which participants were required to reach toward peripheral targets (go-only trials), or during a countermanding task. In the latter case, participants had to perform the same reaches (no-stop trials) as those required in the go-only task unless a stop signal was presented. In this circumstance, participants had to withhold the ongoing movement (stop trials). As expected, the awareness of the presence of the stop signal induced a lengthening of RTs of no-stop trials with respect to those of go-only trials [13]–[17]. However, at the same time, MTs of no-stop trials significantly decreased, probably because the increased length of RTs allowed subjects to fully process movement parameters (e.g. the position of the target) during this epoch. In contrast, in go-only trials the instruction to move as quickly as possible had the effect of speeding up RTs even though some movement parameters had not yet completely computed. Thus it is likely that some additional processing has to take place during action execution, increasing the length of MTs. Clearly this strategy represents an optimization of costs versus benefits because shorter RTs are compensated by longer MTs and vice versa. All in all the study of Mirabella, Pani & Ferraina [13] showed for the first time that proactive control (i.e. a control over response execution in anticipation of known task demands) influences not only the length of the RTs, as already known, but also the duration of the MTs.

Exploiting the same paradigms but with a different aim (i.e. assessing the role of STN in inhibitory control), Mirabella et al. [18] found that RTs of no-stop trials of PD patients were slower than those of go-only trials, but the slowing was not modulated by the DBS state. On the other hand, as the inhibitory control is improved in the DBS-ON condition it was concluded that, at least in this experimental context, STN seems to play a selective role in response inhibition. However, given our previous results [13] we wondered whether, and possibly how, proactive adjustments of MTs could be affected by the DBS.

## Materials and Methods

### Participants

Fifteen participants diagnosed with idiopathic PD, and subsequently treated with bilateral STN DBS complemented by dopaminergic medication, were recruited from the outpatients of the IRCCS Neuromed Hospital.

Patients did not present severe sensory deficits, overt signs of dementia, or severe tremor or rigidity of the right arm. All patients were screened for dementia both before the surgery and before the administration of our task, using the mini–mental state examination (MMSE). On average before surgery the score of the MMSE was higher than just before our examination (29.3±0.35 vs 28.4±0.31; paired t-test t(11) = 2.4, p<0.05). However, in both instances the MMSE values were well above the level indicating cognitive impairments, i.e. 24 [19]. They did not take medication overnight prior to the study and thus, at the time of testing, were in the off-state [20]. All tests were performed 60 minutes after any DBS was switched off or on, so that they were tested in near-steady motor status [21]–[23]. Three patients were unable to complete the study because the bilateral cessation of DBS caused severe impairment of their motor abilities. Clinical features of the remaining patients are reported in Table 1 (see Table S1 in File S1 for a comparison of clinical data of patients immediately before surgery and at the time of the experiment). The surgical technique for implantation of the DBS electrodes has been described previously [18]. Quadripolar electrodes (model 3389; Medtronic Ltd) were used in all patients. Post-surgical reconstruction of the position of the quadripolar lead with respect to STN was performed according to a previously described localization method [24], [25], and locations of active electrode contacts are reported in Table 2.

**Table 1:** Clinical data of PD patients with bilateral implantation of DBS participating in the experiment.

	Age	Sex	Years since diagnosis	Years since surgery	Hoehn & Yahr (ON)	L Dopa eq/Kg	Side of Onset	UPDRS 3			
								ON	ON Right	ON Left	OFF
1	54	M	24	3	1	400	left	15	19	21	24
2	67	M	17	5	1	890	right	30	35	36	43
3	53	F	16	1	1	920	right	21	33	31	50
4	64	F	8	2	2	720	left	34	40	38	35.5
5	53	M	15	5	1	304	right	12	15	17	22.5
6	64	M	22	4	2	920	left	28	36	34	46
7	61	M	17	3	2	1050	right	21	36	35	33
8	64	M	23	4	1	150	right	12	15.5	19	22
9	55	M	22	4	1	640	left	6	14	12	18
10	66	M	16	5	2	640	right	14	30	23	34
11	66	M	20	2	3	450	right	18.5	23.5	20.5	24.5
12	56	M	15	2	3	880	left	16.5	27	25.5	29.5
**Mean** **(SEM)**	**60.2** **(1.6)**		**17.9** **(1.3)**	**3.3** **(0.4)**	**1.7** **(0.2)**	**663.7** **(82.2)**		**19.0** **(2.4)**	**26.0** **(2.4)**	**27.0** **(2.7)**	**31.8** **(2.9)**

For each patient sex, age, years since diagnosis, years since the implantation of the second DBS electrode, Hoehn & Yahr scores (indicating the relative level of disability due to PD disease) in the “ON” phase, L-dopa equivalents/kg, side of onset of symptoms and the Unified Parkinson’s Disease Rating Scale part 3 (UPDRS3, indicating the patient’s motor symptoms) in each DBS condition and without medications, rated by a neurologist (NM) before starting the task, are given.

**Table 2:** For each patient the position of active electrodes, amplitude, pulse and frequency of stimulation are shown separately for the right (R) and the left (L) hemisphere.

Patient	Active electrodes		Amplitude (Volt)		Pulse (microsec)		Frequency (Hz)	
	R	L	R	L	R	L	R	L
1	STNd	STNv^(+)^, STNd ^(−)^*	3.2	3.5	60	60	185	185
2	Zi	Zi	2.1	3.8	90	90	185	185
3	STNd	STNv	3.5	3.3	60	60	185	185
4	Zi	STNd^(+)^, Zi^ (−)^*	3.2	3.5	60	60	185	185
5	STNd	STNv	3.7	3.3	60	60	185	185
6	STNv	Zi	3.6	3.3	60	60	185	185
7	STNv, STNd	STNv, STNv	3.5	3.5	60	60	185	185
8	STNd, STNv	STNv, SNr	3.6	2.6	60	60	185	185
9	STNv	Zi	3.8	4.3	90	90	185	185
10	Zi	STNv	3	3.2	60	60	185	185
11	STNv	STNd	4	4.5	60	60	185	185
12	Zi	STNv	2.6	3.2	60	60	185	185

Active electrodes indicated by a star have a bipolar configuration, with cathode (+) and anode (−); all other electrodes have a monopolar configuration. Abbreviations: SNr, Substantia Nigra pars reticulata; STNd, dorsal subthalamic nucleus; STNv, ventral subthalamic nucleus; Zi, zona incerta.

In addition, 13 healthy control subjects (three females, 10 males; age range 55–68 years; mean age 60.7±1.3 (SEM); *t*-test t(23) = 0.21, p = 0.83) with normal or corrected-to-normal vision were also tested.

All participants were right-handed and signed informed consent in accordance with the Declaration of Helsinki of 1964, and all procedures were approved by the local ethics board of the Neuromed hospital.

### Apparatus

Subjects were seated in a darkened and silent room, in front of a 17-inch PC monitor (LCD, refresh rate 75 Hz, 640×480 resolution) on which visual stimuli, consisting of red circles (2.4 cd/m^2^) with a diameter of 2.8 cm against a dark background of uniform luminance (<0.01 cd/m^2^), were presented. The PC monitor was equipped with a touch screen (MicroTouch; sampling rate 200 Hz) for touch-position monitoring. A non-commercial software package, CORTEX, was used to control stimulus presentation and to collect behavioral responses. The temporal arrangements of stimulus presentation were synchronized with the monitor refresh rate.

### Behavioral Tasks

**Figure 1:**
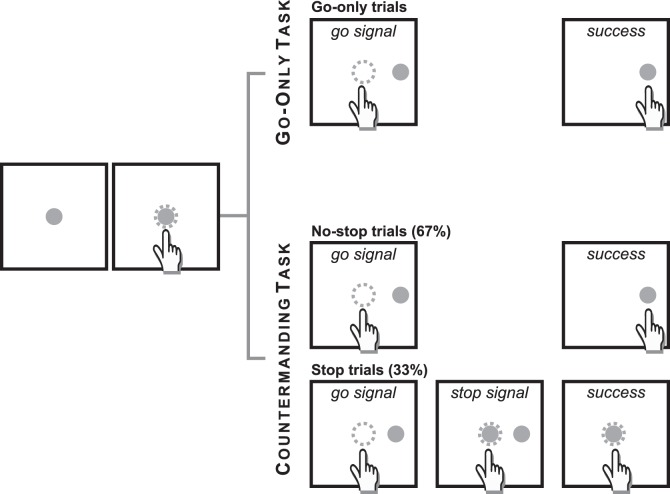
Temporal sequence of the visual displays for no-stop and stop trials in the countermanding reaching task. Temporal sequence of the visual displays for each task. All trials began with the appearance of a central stimulus. The subject had to reach and hold it with the index of the right (dominant) hand for a variable period of 500–800 ms. In the go-only task and in the no-stop trials of the countermanding task, the central stimulus disappeared and, simultaneously, a target appeared to the right, acting as a go-signal. Subjects were instructed to perform a speeded reaching movement toward the peripheral target. Randomly, in the 33% of trials of the countermanding task (stop trials), the central stimulus (stop signal) reappeared at variable delays after the go signal (SSDs), indicating that the subject should cancel the pending movement. If subjects executed the reaching movement the trial was scored as a stop-failure trial (not shown). The dotted circle (which was not visible to the subjects) indicates the size of the tolerance window for the touches (3.5 cm diameter).

Participants performed, always with the right (dominant) arm, similar reaching movements in two different cognitive paradigms : a) a go-only task and b) a countermanding task ([13], [26]; Fig. 1). The two tasks were presented in separate blocks and the order of presentation was counterbalanced across participants. Resting periods were allowed between blocks whenever requested.

All trials began with the appearance of a stimulus at the center of the display and subjects were required to touch and hold it for 500–800 ms. Thereafter, in go-only trials, the central stimulus disappeared and, simultaneously, a red circle target appeared (go-signal) at 15 cm to the right of the central stimulus. To make a correct response, subjects had to perform a speeded reaching movement toward the peripheral target and hold it for 300 ms (Fig. 1).

The countermanding task (Fig. 1) consisted of a random mix of 67% no-stop trials and 33% stop trials. No-stop trials were identical to go-only-trials. Stop trials differed from the no-stop-trials because at a variable delay (stop signal delay; SSD) after the presentation of the go-signal the central stimulus reappeared (stop signal), indicating that subjects should inhibit their movements. Trials in which subjects successfully withheld the movement were defined as stop-success trials and those in which they moved were defined as stop-failure trials. Auditory feedback was given for correct responses and error trials were not repeated.

The length of the SSDs was dynamically changed using a staircase procedure [27]–[29] in order to allow participants to succeed in cancelling the response in about 50% of the stop trials. The SSD duration varied from one stop trial to the next according to the behavioral performance: if subjects succeeded in halting their response then the SSD increased by three refresh rates (39.9 ms); otherwise the SSD decreased by the same amount of time. The staircase started from an SSD of 119.7 ms. Both the incremental step and the starting point of the staircase have been chosen following pilot studies which indicated that these values were appropriate for quickly obtaining the desired amount of inhibition in stop trials.

Before starting the countermanding task, subjects were informed that in some stop trials they would not be able to cancel the movement and that they should not be troubled by this. We stressed the importance of reaching the peripheral target as fast as possible. In addition, to discourage participants from slowing down responses in order to cancel their movements more easily, a) we verbally informed them that the probability of stopping would approximate to 50%, irrespective of whether they were postponing their response or not; b) we set an upper RT limit for no-stop trials which meant that, whenever RTs were longer than 800 ms, no-stop trials were signaled as errors during task performance but were kept for the final analysis (overreach trials).We adopted this strategy because we wanted to put a time pressure on participants, but we did not want to truncate the distributions of the RTs. To avoid this inconvenience we allowed subjects to wait longer than 800 ms but as soon as they detached their index finger from the touchscreen we aborted the trial. Parkinson’s patients performed the tasks in four experimental conditions: a) both DBS-OFF; b) both DBS-ON; c) DBS-L-ON; d) DBS-R-ON. Stimulation conditions were counterbalanced across patients and administered in two different experimental sessions occurring on different days. In each condition patients were required to complete four blocks of 60 trials (240 trials); overall each subject performed 960 trials. Before starting the task, about 50 practice trials were given for familiarizing subjects with the apparatus. Age-matched healthy subjects performed a single countermanding block consisting of 240 trials. In addition, all participants were required to complete one block of 75 go-only trials.

### Data Analysis

As all salient results about movement inhibition are perfectly in line with those of Mirabella et al. [18] and they are not relevant for the present paper, we will not deal with them (with respect to the previous paper we added two more patients).

We defined the RT as the time elapsing from the appearance of the go-signal to the finger detaching from the touch-screen. MT was defined as the elapsed time between the detaching from the central target and touching the peripheral target.

Whenever ANOVAs were employed, Mauchley’s test evaluated the sphericity assumption and, where appropriate, correction of the degrees of freedom was made according to the Greenhouse–Geisser procedure. When needed, *post hoc* tests (pairwise comparisons) with Bonferroni correction were employed.

## Results

### Effectiveness of DBS on Motor Symptoms

Motor symptoms in each DBS condition and without medication were scored exploiting the Unified Parkinson’s Disease Rating Scale part 3 (UPDRS3). To determine whether and when STN DBS produced significant improvements we ran a one-way repeated-measures ANOVA with DBS condition as factor. We found a main effect (F[1.8] = 25.8, p<0.001). Pairwise comparisons demonstrated that the UPDRS3 score was significantly lower in the DBS-ON condition than in all the other conditions (ps<0.001) and that the UPDRS3 in the DBS-R-ON condition was lower than in the DBS-OFF condition (p<0.05).

### Adaptation of RTs and MTs to the Experimental Context

**Figure 2:**
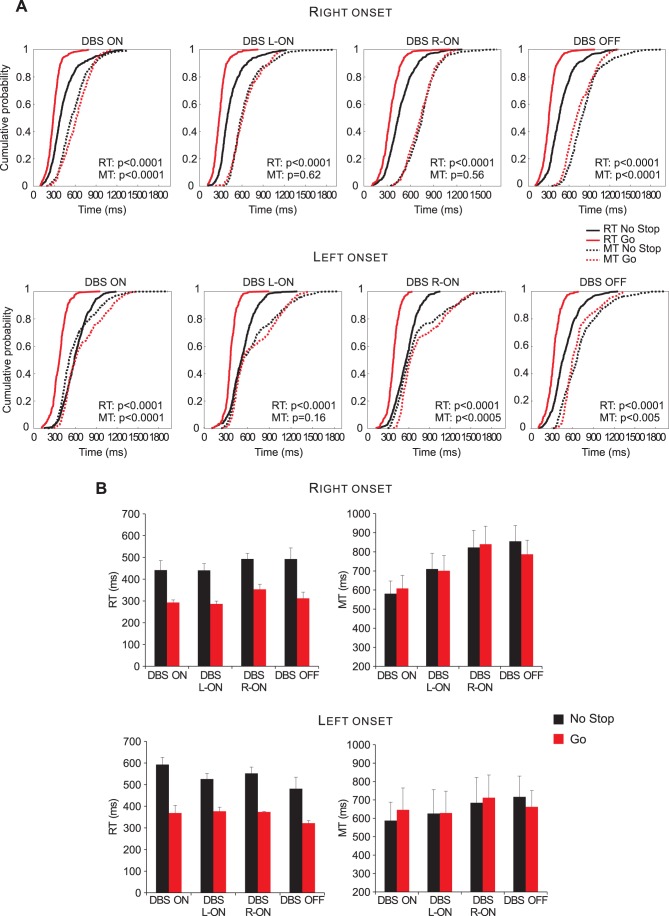
Reaction times (RTs) and movement times (MTs) for reaching movements in Parkinson’s patients with right- and left-side body onset of the disease. A. Cumulative distributions of RTs and MTs. These distributions were obtained by considering separately, according to laterality of onset of motor symptoms, the single RTs and MTs of no-stop and of go-only trials of subjects with right-body onset(n = 7; upper row) with left-body onset of the disease (n = 5; lower row). Bold lines represent RTs, dotted lines represent MTs. For each condition the p-value of Kolmogorov–Smirnov test is given, both for RTs and for MTs. B. Histograms of average RTs (upper row) and of MTs (lower row) of no-stop and go-only trials in each DBS condition. Bars represent the standard error of the mean.

It is known that PD symptoms usually become apparent primarily on one side of the body (e.g. [30]) and this motor symptom asymmetry is associated with asymmetric dopaminergic degeneration in the brain (e.g. [31]). Given that it has been hypothesized that, in humans, voluntary inhibition of arm movements relies upon a right-lateralized frontal–basal ganglia–thalamic pathway [32], as a first step in our analyses we split our patients according to the side of onset of symptoms. To assess whether the asymmetric nigral cell loss produced differential effects on a patient’s performance, we plotted the cumulative distributions of RTs and of MTs of go-only versus no-stop trials separately for right-onset (n = 7) and for left-onset PD patients (n = 5). As shown in Fig. 2, the two groups of patients had similar behavior in the DBS-ON condition (patients always exhibited longer RTs and shorter MTs in no-stop trials than in go-only trials) and in the DBS-OFF condition (patients always had longer RTs and MTs in no-stop trials than in go-only trials). In the DBS-L-ON and DBS-R-ON conditions, while the lengthening of RTs in no-stop trials was always significant, MTs showed different patterns. Clearly in right-onset PD patients, MTs of no-stop-trials did not differ from those recorded during go-only trials. However, in left-onset patients in both DBS condition there was a tendency for MTs of no-stop trials to be shorter than MTs of go-only trials. This tendency reached significance for the DBS-R-ON condition but not for the DBS-L-ON condition. Nevertheless, these results suggest that a unilateral stimulation of STN might produce a different effect according to the side of onset disease.

An obvious limitation of the present analysis is the low number of patients within each group, so further studies are needed to confirm this finding. As a consequence, in the following sections we will report and discuss only the results for the two conditions (i.e. DBS-ON and DBS-OFF) in which patients exhibited congruent behavioral patterns.

**Figure 3:**
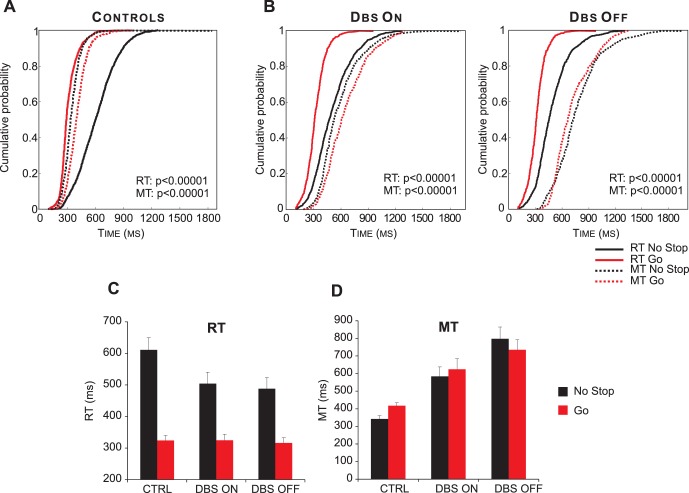
Reaction times (RTs) and movement times (MTs) for reaching movements across the populations of Parkinson’s patients and control subjects. A. Cumulative distribution of RTs (solid lines) and MTs (dotted lines) of healthy subjects (n = 13) for go-only (grey) and no-stop trials (black). B. Cumulative distributions of RTs (solid lines) and MTs (dotted lines) of DBS patients (n = 12) in DBS-ON and DBF-OFF conditions for both go-only (grey lines) and no-stop (black lines) trials. For each condition the p-value of Kolmogorov-Smirnov test is given, both for RTs and for MTs. C. Histograms of average RTs of no-stop and go-only trials in DBS-ON and DBF-OFF conditions. Bars represent the standard error of the mean. D. Histograms of average MTs of no-stop and go-only trials in each DBS-ON and DBF-OFF conditions. Bars represent the standard error of the mean.

Figure 3 and Table 3 illustrate the main findings of the present study. Data from single participants are combined to create cumulative distributions of RTs and of MTs of go-only versus no-stop trials at the population level (Fig. 3A and B). As expected, the two experimental paradigms had opposite effects on RTs and MTs of healthy controls [13]. In fact, they exhibited longer RTs and shorter MTs in no-stop trials than in go-only trials (Fig. 3A; Kolmogorov–Smirnov test, p*s*<0.0001). Qualitatively, PD patients in the DBS-ON condition showed a similar pattern (Kolmogorov–Smirnov test, p*s*<0.0001; Fig. 3B, left panels). However, in the DBS-OFF condition both RTs and MTs were longer in no-stop trials than in go-only trials (Kolmogorov–Smirnov test, p*s*<0.0001; Fig. 3B, right panel).

**Table 3:** Summary of behavioral measurements for Parkinson’s patients in each DBS condition and for healthy controls in no-stop trials and in go-only trials.

	DBS-ON	DBS-OFF	Age- Matched CTRL
RT no-stop trials (ms)	504.3±36.1	487.9±35.4	611±38.6
RT go-only trials (ms)	324.6±19.1	315.9±17.1	324.1±15.7
MT no-stop trials (ms)	583.9±53.9	797.3±67.2	342.2±18.9
MT go-only trials (ms)	623.9±60.7	734.9±57.2	417.7±16.3
RT (Percentage difference)	57.8±10.9	54.29±6.9	89.7±9.3
MT (Percentage difference)	−5.5±3.1	8.9±4.5	−17.9±3.6
Accuracy in no-stop trials	0.86±0.03	0.89±0.03	0.86±0.03
Accuracy in go-only trials	0.93±0.02	0.90±0.03	0.97±0.01
Percentage of overreaches among no-stop trials	0.06±0.02	0.03±0.01	0.1±0.003
Percentage of overreaches among go-only trials	0.001±0.001	0.003±0.002	0.003±0.002

In all cases the average values ±SEM are reported. Accuracy was computed as the percentage of correct responses with respect to the sum of correct plus incorrect responses. Incorrect trials were either those in which subjects touched the target outside the tolerance window or those whose RTs exceeded the upper RT (overreach trials). The percentage of overreach trials was computed with respect to the sum of correct plus incorrect responses.

A two-way repeated-measures-ANOVA (factors: DBS condition and RT no-stop trials/go-only trials) showed that, on average, RTs of PD patients were shorter when executing go-only trials than when executing no-stop trials (RT no-stop trials/go-only trials: F [1], [11] = 67.7, p<0.0001; see Fig. 3C). However, RTs did not differ across DBS conditions (F [1], [11] = 0.31, p = 0.59; interaction was also not significant: F [1], [11] = 0.43, p = 0.84). The same analysis performed on MTs (two-way repeated-measures-ANOVA; factors: DBS condition and MT no-stop trials/go-only trials; see also Fig. 3D) revealed that MTs of no-stop and go-only trials were not different (MT no-stop trials/go-only trials, F [1], [11] = 0.39, p = 0.54), but they differed according to the DBS state (DBS condition, F [1], [11] = 10.3, p<0.01). The interaction (F [1], [11] = 6.54, p<0.05) better explained this result, revealing that while MTs of go-only trials did not differ between the DBS conditions, MTs of no-stop-trials were significantly faster in the DBS-ON than in DBS-OFF condition (pairwise comparisons, p<0.05).

RTs and MTs of control subjects were compared with those of PD patients in each DBS condition, using four two-way-ANOVAs with group (control subjects and PD patients in a given DBS state) and trial type (go-only and no-stop trials) as factors. As reported in Table 4, RTs of go-only trials were always longer than those of no-stop trials. Additionally we also found that RTs of no-stop trials were longer in control subjects than in PD patients both in the DBS-ON and DBS-OFF conditions (pairwise comparisons, p*s*<0.05). The comparison of MTs between controls and PD patients in the DBS-ON condition showed that i) the former were overall faster than the latter, ii) MTs of go-only trials were shorter than those of no-stop trials. Also PD patients with DBS OFF had longer MTs than healthy participants. However, while controls had longer MTs in go-only trials than in no-stop trials (pairwise comparisons, p<0.01), PD patients with DBS OFF showed the opposite relationship, i.e. MTs in no stop trials were longer than those of go-only trials (pairwise comparisons, p<0.05).

**Table 4:** Results of the two-way ANOVAs on the RTs and MTs with group (control subjects, CTRL, versus PD patients in each DBS state) and trial type (go-only versus no-stop trials) as factors.

	Group		Trial type		Group*Trial type	
	F(df)	Sig.	F(df)	Sig.	F(df)	Sig.
RTs CTRL vs. DBS-ON	F(1,23) = 2.3	0.14	F(1,23) = 110.6	<0.0001	F(1,23) = 5.8	<0.05
RTs CTRL vs. DBS-OFF	F(1,23) = 3.3	0.08	F(1,23) = 138.5	<0.0001	F(1,23) = 8.7	<0.01
MTs CTRL vs. DBS-ON	F(1,23) = 15.4	<0.001	F(1,23) = 26.9	<0.0001	F(1,23) = 2.5	0.12
MTs CTRL vs. DBS-OFF	F(1,23) = 41.7	<0.0001	F(1,23) = 0.13	0.73	F(1,23) = 13.9	<0.001

*F-*values, degrees of freedom (df) and significance value (Sig.) are reported for both main factors and interactions.

**Figure 4:**
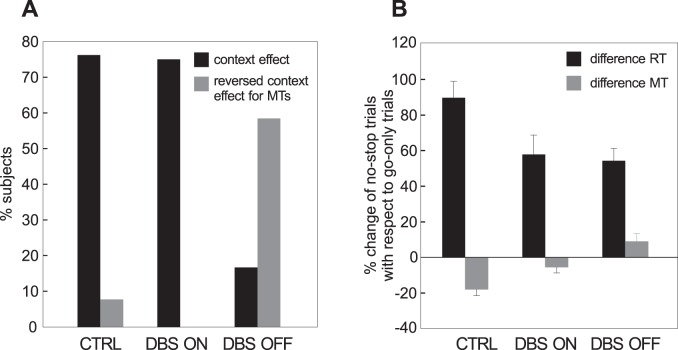
Magnitude and modulation of the context effect. (see Results for more details) **A.** Percentage of PD patients (in each DBS state) and healthy subjects showing simultaneously a significant increase in reaction times (RTs) and a significant decrease in movements times (MTs) in no-stop trials with respect to go-only-trials (black bars), and a significant lengthening of both RTs and MTs in no-stop-trials with respect to go-only trials (gray bars). **B.** Percentage change (±SEM) for both RTs (black bars) and MTs (grey bars) of no-stop-trials with respect to go-only trials in PD patients (in each DBS state) and control subjects.

In order to take into account the contribution of each subject, we considered how many participants exhibited a simultaneous decrease in RTs and increase in MTs in no-stop trials with respect to go-only trials (‘context effect’). As shown in Fig. 4A, 10 out of 13 controls (76.9%) exhibited a context effect. In one control (7.7%), RTs and MTs were simultaneously longer in no-stop trials (‘reversed MT effect’). In the DBS-OFF condition seven out of 12 (58.3%) patients had both RTs and MTs longer in no-stop trials, while only two of them (16.7%) had a context effect. This picture changed dramatically in the DBS-ON condition: 75% of patients (nine out of 12) showed a context effect.

To quantify the relative changes in RTs and MTs across the two experimental paradigms, we computed the percentage difference of the average values of these two parameters in no-stop trials with respect to go-only-trials (Table 3), from the following formula:



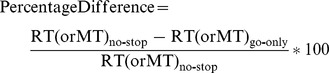



Positive values indicate an increase in a given parameter in no-stop trials with respect to go-only-trials, and vice versa.

The change in RTs was much larger than that in MTs, both in controls and in PD patients independently of the DBS state (Fig. 4B). The amount of increase in RTs in no-stop-trials across DBS conditions was the same (paired t-test, t [11] = 0.24, p = 0.81). In contrast, the MT percentage change across the DBS conditions was significant (paired t-test t [11] = 2.42, p<0.05).

Next, we compared the RT percentage change in control subjects with the corresponding values at each DBS condition using t-tests. We found that the percentage increase in RTs in no-stop trials with respect to go-only trials of healthy participants were larger than in all DBS conditions (DBS-OFF, t [23] = 3, p<0.01; DBS-ON, t [23] = 2.2, p<0.05). Similarly, the MT percentage decrease was also always larger in healthy subjects than in PD patients (DBS-OFF, t [23] = 4.6, p<0.0001; DBS-ON, t [23] = 2.57, p<0.05).

Finally, in order to check whether there was a relationship between the clinical evaluation of motor symptoms and the behavioral parameters, we measured RTs and MTs of no-stop trials and go-only trials and computed separate linear correlations of the RTs and MTs with the UPDRS3 score of each patient. The correlations were never significant (see Fig. S1 in File S1).

### Accuracy

As it is known that faster MTs result in a loss of accuracy [33] we estimated the percentage of correct responses of PD patients in each DBS condition and for each trial type (Table 3). As we did not found any significant effect (two-way repeated-measures-ANOVA, factors: DBS conditions F [1], [11] = 0.21, p = 0.66; trial type, F [1], [11] = 2.91, p = 0.12; interaction, F [1], [11] = 1.32, p = 0.28), we concluded that accuracies were very similar across experimental conditions.

Finally we compared the accuracy of control subjects with that of PD patients in each DBS condition with the corresponding values at each DBS condition, using two-way-ANOVAs with group (control subjects and PD patients in a given DBS state) and trial type (go-only and no-stop trials) as factors. As reported in Table 5, the accuracy of healthy subjects was never significantly different from that of PD patients. However, subjects were more accurate when performing go-only trials than no-stop trials.

**Table 5:** Results of the two-way ANOVAs on the accuracy with group (control subjects, CTRL, versus PD patients in each DBS state) and trial type (go-only versus no-stop trials) as factors.

	Group		Trial type		Group*Trial type	
	F(df)	Sig.	F(df)	Sig.	F(df)	Sig.
CTRL vs. DBS-ON	F(1,23) = 0.3	0.58	F(1,23) = 17.5	<0.0001	F(1,23) = 1.3	0.26
CTRL vs. DBS-OFF	F(1,23) = 0.7	0.42	F(1,23) = 8.7	<0.01	F(1,23) = 3.4	0.07

*F-*values, degrees of freedom (df) and significance value (Sig.) are reported for both main factors and interactions.

## Discussion

We have shown for the first time, to our knowledge, that the bilateral stimulation of STN can partially restore a planning strategy appropriate to the context in which PD patients are embedded, i.e. qualitatively similar to that of healthy subjects [13]. We compared similar arm reaching movements executed in a go-only task with those executed in a countermanding task. In both cases we stressed the importance of moving as fast as possible, but subjects were aware that in the latter task a stop signal could sometimes appear during movement preparation, indicating that the pending action should be suppressed. The presence of the stop signal creates a conflict on all no-stop trials because, despite the instructions, subjects spontaneously tend to delay movement initiation to wait for the possible occurrence of a stop signal. In healthy subjects this conflict had opposing effects on RTs and MTs of reaching movements when compared with a situation in which similar movements were performed in a task without intermingled stop signals [13]. Probably the delaying of no-stop movement initiation has the side-effect of allowing the coding of a full motor program, speeding up MTs. In the go-only trials the absence of a brake allows a shortening of RTs at the cost of leaving some details of the motor program uncompleted, so that the planning must be completed during the movement. Data collected both on healthy subjects [11], [13], [34], [35] and on PD patients [12] support the idea that an action plan can be updated beyond the initiation phase.

In our opinion turning off both DBS decreases the ability to orchestrate movements according to the context in which PD patients act. It has been shown that PD patients, either with or without dopaminergic medication, fail to exhibit appropriate context-dependent adjustments to standing postural control aimed at reducing the risk of a fall when their balance is challenged by a threat [36], [37]. In particular, we believe that the absence of STN stimulation might cause an improper evaluation of movement energy costs. Recently, Mazzoni, Hristova & Krakauer [38] reached a similar conclusions. They asked PD patients and healthy controls to move their arm to a previously specified target at different speeds a given number of times. Both groups were able to make the required movements with the same accuracy, but PD patients had to make significantly more trials before reaching the required number of repetitions. As the accuracy of patients was the same as that of controls, Mazzoni, Hristova & Krakauer [38] concluded that the loss of dopamine did not cause bradykinesia through a speed–accuracy trade-off, but rather affected decision-making through a faulty evaluation of the costs of movements. This interpretation might be valid even in our experiments, as we also did not find a difference in no-stop trial accuracy across DBS conditions. It is likely that these effects occur because DBS switches off pathological activity in the STN and imposes a new type of discharge endowed with beneficial influences [39].

### Neural Basis of Contextual Effects

Previously we found that STN DBS improved inhibitory control but did not affect RTs of reaching movements [18]. Thus we suggested that STN seemed to play a selective role in response inhibition. Now, as we have found a modulation of MTs as a function of the DBS state, we can extend our previous conclusions to include this new finding.

The role of the STN and the mechanisms through which DBS exerts clinical effects are hotly debated. In fact, while DBS of STN seems to improve volitional inhibition ([18], [40], [41] but see [42] for a different view) it has also been shown to increase impulsive behavior [43], [44]. These paradoxically discrepant findings might be explained by impulse and inhibitory control underlying goal-directed behavior being regulated by two different circuits which both include the STN, impulse control passing through the pre-SMA/SMA [45] and inhibitory control passing through the right IFG [32], [45]. Support for this view comes from the study by Wylie et al [46], who administrated a version of the Simon task to PD patients with STN DBS. Patients were asked to make left or right key-presses to the appearance of a blue or a green circle, respectively (relevant stimulus dimension). Circles could be presented either in the left or right visual field (irrelevant stimulus dimension). As expected, irrelevant stimulus information elicited a strong response impulse which interfered with the response when it was incongruent with the relevant stimulus information (i.e. when a blue circle appeared in the right visual field) and vice versa. Wylie et al. [46] analyzed both the accuracy and size of the Simon effect (i.e. the difference in the response speed between incongruent and congruent trials) separately at each bin of the RT distribution. They found that, in the fastest bins, patients were more accurate in the DBS-OFF than the DBS-ON condition, indicating that STN stimulation increased the impulsiveness of the fastest responses. However, in the slowest bins STN stimulation significantly reduced the Simon effect, indicating that the inhibitory control needed to suppress response impulses driven by the irrelevant stimulus dimension was improved. Thus it is possible that, in our task conditions, bilateral STN DBS might simultaneously improve inhibitory control and strategic planning in a context-dependent manner acting through parallel circuits.

A similar context-dependent effect has been observed in the oculomotor system. Stuphorn & Schall [47] showed that microstimulation of the supplementary eye fields of behaving monkeys influences saccade generation in an adaptive way. The delivery of subthreshold microstimulation during no-stop trials delayed the saccade generation, while the same stimulation reduced RTs in the context of a visually guided saccade task (i.e. in blocks without stop signals). This is adaptive because, in the context of a countermanding task, slower saccades have a higher probability of being cancelled if a stop signal is presented, while in the context of a go-only task faster saccades allowed the monkeys to quickly obtain juice rewards. The fact that in our case the stimulation influenced MTs instead of RTs might be due to the different nature of reaching movements and saccades. Saccades are ballistic movements, i.e. they have a “point of no return” after which movement preparation is no longer controllable. Different arm movements can be stopped at any point along their path [48], [49] and, as a consequence, their planning can be modified even during the execution phase.

Interestingly, STN DBS does not affect the amount of lengthening of RTs of no-stop-trials with respect to those of go-only-trials [18]. However, slowing down of no-stop trials is altered by PD, given that patients react faster than age-matched controls. Evidently this form of proactive control depends upon damage to the striatum and/or to the connected circuitry, which are known to be critical for this function [50], [51]. At the same time our data reveal that another aspect of proactive control which takes place during the countermanding task, namely the shortening of MTs of no-stop-trials, is affected by STN DBS. Therefore it is possible that overall proactive preparation of actions relies on two different circuits, one passing through the striatum and the other passing through STN.

## Supporting Information

File S1 Supporting table and figure.
(DOC)
