# Supplementary material for: Stimulation of Subthalamic Nuclei Restores a Near Normal Planning Strategy in Parkinson’s Patients
Source: PLoS One. 2013 May 3;8(5):e62793. doi: 10.1371/journal.pone.0062793 (PMC3643906; doi:10.1371/journal.pone.0062793)
Supplement: File S1 — Supporting table and figure. (DOC) [file pone.0062793.s001.doc]

**SUPPLEMENTARY MATERIALS**

|  | **UPDRS 3 pre-surgery** | |  | **UPDRS 3**  **post-surgery** | | | |  | **L-dopa equivalent pre surgery** | **L-dopa equivalent post surgery** |  | **percentage of fluctuations reduction** | **percentage of diskynesias reduction** |  | **Reason of surgery** |
| --- | --- | --- | --- | --- | --- | --- | --- | --- | --- | --- | --- | --- | --- | --- | --- |
|  | Med On | Med Off |  | MedOn DBSOn | MedOn DBSOff | MedOff DBSOn | MedOff DBSOff |  |  |  |  |  |  |  |  |
| **1** | surgery elsewhere | |  | 11 | 22 | 15 | 24 |  | - | 400 |  | surgery elsewhere | |  | motor fluctuations, dyskinesias |
| **2** | 18 | 32 |  | 8 | 22 | 30 | 43 |  | 236 | 890 |  | 90% | 90% |  | dyskinesias, daytime sleepiness, confusion related to apomorphine |
| **3** | 21 | 51 |  | 18 | 24 | 21 | 50 |  | 730 | 920 |  | 60% | 60% |  | motor fluctuations, dyskinesias |
| **4** | 29 | 51 |  | 14 | 34 | 34 | 35.5 |  | 910 | 720 |  | 70% | 70% ***** |  | dyskinesias, daytime sleepiness, confusion related to apomorphine, worsening camptocormia |
| **5** | 25 | 50 |  | 10 | 27 | 12 | 22.5 |  | 820 | 304 |  | 80% | 90% |  | motor fluctuations, dyskinesia, dystonia of the lower limbs |
| **6** | 28 | 48 |  | 19 | 26 | 28 | 46 |  | 1022 | 920 |  | 60% | 60% |  | motor fluctuations, dyskinesias daytime sleepiness |
| **7** | 13 | 36 |  | 16 | 21 | 21 | 33 |  | 566.5 | 1050 |  | 70% | 80% ***** |  | dyskinesias, daytime sleepiness, confusion related to apomorphine, worsening camptocormia |
| **8** | 29 | 60 |  | 12 | 25 | 12 | 22 |  | 498 | 150 |  | 100% | 100% |  | motor fluctuations, dyskinesias |
| **9** | 27 | 47 |  | 13 | 16 | 6 | 18 |  | 1040 | 640 |  | 80% | no diskynesias |  | no tremor responsiveness to medical therapy |
| **10** | 33 | 62 |  | 14 | 44 | 14 | 34 |  | 492 | 640 |  | 70% | 70% |  | motor fluctuations, dyskinesias |
| **11** | 19 | 34 |  | 14 | 19 | 18.5 | 24.5 |  | 788 | 450 |  | 70% | 90% |  | motor fluctuations, dyskinesias |
| **12** | surgery elsewhere | |  | 13 | 25 | 16.5 | 29.5 |  | - | 880 |  | surgery elsewhere | |  | motor fluctuations, dyskinesias |
| **Mean**  **(±SEM)** | **24.20**  ±1.8 | **47.10**±2.9 |  | **13.50**  ±0.9 | **25.42**  ±2.1 | **19.00**  ±2.4 | **31.83**  ±2.9 |  | **710.25**  ±74.7 | **663.67**  ±82.2 |  |  |  |  |  |

* still camptocormic

**Table S1.** C**linical data of patients** **immediately before surgery and at the time of the experiment.** For each patient, UPDRS part3 before and after surgery, L-dopa equivalents, percentage of motor fluctuation, dyskinesias reduction after surgery and the reasons why patient underwent surgery are given.

1. **Correlation between UPDRS3 and behavioral parameters**

We tried to relate all our behavioral measures (RTs/MTs of no-stop trials; RTs/MTs of go-only trials) with the UPDRS3 score of each patient (figure S1). Linear correlation was never significant and the slopes of the regression lines did not have the same sign, sometimes were positive, some other times were negative. Thus it seems that these parameters might not necessarily represent the best behavioral measure to describe motor improvements measured with the UPDRS3 scale. Clearly a serious limitation to these conclusions is given by the small number of patients. However, the items of the UPDRS3 scores several different motor skills (e.g. coordination, postural adjustments, balance) and therefore the improvements might come from an amelioration of these aspects of the motor function that were not measured in our experiments.


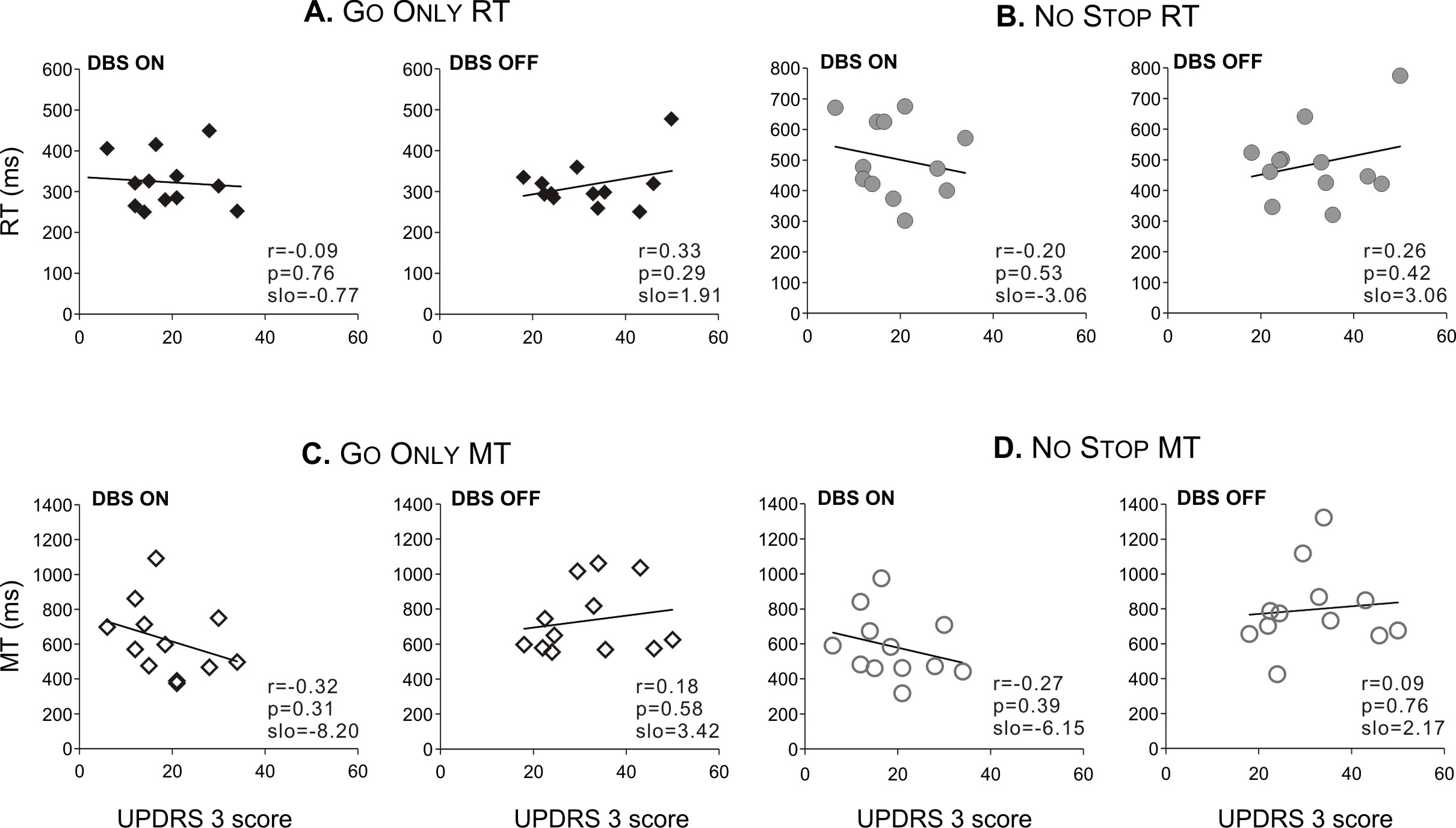


**Figure S1.** Scatter plots with regression line between UPDRS3 score and go-only trials RTs (panel A), no-stop trials RTs (panel B); go-only trials MT (panel C); no-stop trials MTs (panel D) in each DBS condition. Each dot represents the average value of a given behavioral parameter for each PD patient. In each graph, the Pearson correlation coefficient (r), the corresponding relative p-values and the angular coefficient of the regression line (slo) are shown. Normality tests of the distributions (Shapiro-Wilk test) showed that in all cases, but one (RTs of go-only trials, DBS-OFF condition), data were normally distributed.
